# The Breakup of a Helium Cluster After Removing Attractive Interaction Among a Significant Number of Atoms in the Cluster

**DOI:** 10.1038/s41598-020-62732-2

**Published:** 2020-04-01

**Authors:** Tao Pang

**Affiliations:** Department of Physics and Astronomy, University of Nevada, Las Vegas, Nevada 89154-4002 USA

**Keywords:** Quantum fluids and solids, Quantum simulation

## Abstract

The breakup of a quantum liquid droplet is examined through a ^4^He cluster by removing the attractive tail in the interaction between some of the atoms in the system with the diffusion quantum Monte Carlo simulation. The ground-state energy, kinetic energy, cluster size, and density profile of the cluster are evaluated against the percentage of the atoms without the attractive tail. The condition for the cluster to lose its ability to form a quantum liquid droplet at zero temperature is found and analyzed. The cluster is no longer able to form a quantum liquid droplet when about two-thirds of pairs of attractive interaction are removed. The findings are helpful to the current studies on the formation of quantum liquid droplets from cold atoms.

## Introduction

Recent research in cold atoms has found that by switching the interaction between some of the atoms from being repulsive to attractive, the system can form a quantum liquid droplet^1,2^, supporting the prediction from a mean-field theory^3^. Knowledge beyond mean-field theory about this phase transition from a gas to a liquid in cold atoms is still lacking. The breakup of a cold atom cluster with pure repulsive interaction after the removal of the trapping potential has clearly been seen in earlier experiments^4,5^. However, there is another route to form a quantum liquid droplet in a cluster of quantum particles through the three-body correlation, an outcome of the Efimov effect^6,7^. These theoretical and experimental studies are significant in understanding the behavior of a quantum many-body system at low temperature. The issue, however, is far from being resolved because a mean-field treatment is not enough to include all the correlation effect and does not provide precise information about the system or the correlation between the particles. Further theoretical studies that eliminate any approximation are keenly sought at the moment.

A system related to cold atoms is a cluster of helium atoms that is a quantum liquid droplet at zero temperature even though the size of the cluster can be quite small^8,9^. The van der Waals force between any two helium atoms creates an attractive tail in the helium–helium interaction and stabilizes cluster against quantum fluctuation in the system. Detailed calculations have shown that collective excitations are formed in helium liquids and solids^10^ and small helium clusters are stable quantum liquid droplets^9^. The two systems, cold atoms and helium clusters, are so different in density and interaction, but yet similar in association with quantum fluctuation and competing contributions in interaction^8^. In this work, we wish to answer some of the questions associated with the formation of a quantum liquid droplet in a quantum cluster by examining the role played by the attractive part of the atom–atom interaction in a ^4^He cluster closely. We know for a fact that the attractive tail is essential for a helium cluster to be a quantum liquid droplet at zero temperature, overcoming the strong quantum fluctuation showing in the cluster, even when the cluster size is very small, for example, with less than a hundred atoms.

Here we remove the attractive tail in the atom–atom interaction in a cluster of ^4^He atoms one by one and evaluate the properties of the cluster and their changes without making any approximation in the calculation so that we can have the precise knowledge of the system against the removal of the attractive tail. The first quantity that we concentrate on is the ground-state energy of the cluster, which provides an overall behavior of the system. For a quantum liquid droplet, we expect that the atoms are bound to the center of mass of the cluster and thus has a ground-state energy below zero. To analyze the role played by the motion of the atoms in the cluster, we also calculate the change of kinetic energy of the cluster alongside with the ground-state energy. The kinetic-energy change provides knowledge of the dynamics and quantum fluctuations of the particles in the system. Furthermore, we study the size and the shape of the cluster influenced by the removal of the attractive tail in the interaction. The findings reported here are significant in understanding the formation of a quantum liquid droplet and may shed some light on the current studies on the formation of quantum liquid droplets from cold atoms.

## Model Hamiltonian and simulation method

A general many-body system of *N* particles, each of mass *m*, is described by Hamiltonian

1 $$H={H}_{0}+{V}_{{\rm{I}}},$$

where *H*_0_ is the Hamiltonian for the corresponding noninteracting system:

2 $${H}_{0}=-\frac{{\hslash }^{2}}{2m}\mathop{\sum }\limits_{i=1}^{N}{{\rm{\nabla }}}_{i}^{2}+\mathop{\sum }\limits_{i=1}^{N}{U}_{{\rm{ext}}}({{\bf{r}}}_{i}),$$

with the first term being the total kinetic energy of the system and *U*_ext_(**r**_*i*_) being the external potential energy of the *i*th particle at position **r**_*i*_ in space. The interaction energy *V*_I_ can be quite complex in general, but relatively simple if there is only a pairwise interaction between any two particles in the system:

3 $${V}_{{\rm{I}}}=\mathop{\sum }\limits_{i > j=1}^{N}V({r}_{ij}),$$

with *V*(*r*_*ij*_) being the interaction energy between the *i*th and *j*th particles, depending only on the separation distance of the two particles, *r*_*ij*_ = |**r**_*j*_ − **r**_*i*_|. The external potential energy *U*_ext_(**r**_*i*_) = 0 if the cluster is free in space, namely, not near any other substance. Extensive efforts have been made in finding an accurate representation of the pairwise interaction between two helium atoms, including different fittings from first-principles calculations. The most popular parameterized pairwise potentials between two helium atoms are a version of the Aziz potential known as HFD-B3-FCI1^11^ and the TTY potential^12^. We will use HFD-B3-FCI1 to test our simulation code before using it with a traditional Lennard–Jones potential, a version used by McMillan in the very first variational quantum Monte Carlo study of helium liquids^13^.

### Diffusion quantum Monte Carlo

To calculate the ground-state properties of the many-body system, we here use the standard diffusion quantum Monte Carlo method^14^, which is exact for a many-body Bose system with a controllable variance. The method, with some relevant details, is outlined here. We can rewrite the time-dependent Schrödinger equation into a diffusion equation by replacing the time *t* with an imaginary *τ* = *it*/*ℏ*:

4 $$\frac{\partial \Psi ({\bf{R}},\tau )}{\partial \tau }=-(H-{E}_{{\rm{R}}})\Psi ({\bf{R}},\tau ),$$

where **R** = (**r**_1_, **r**_2_, …, **r**_*N*_) is used to represent the positions of all the particles in the system and *E*_R_ is a reference energy adjustable in the simulation and used to speed up the convergence during sampling.

A good starting point is to have Ψ(**R**, 0) = Φ(**R**), the trial wavefunction that has been optimized through a variational quantum Monte Carlo simulation. We can multiply the imaginary-time Schrödinger equation by Φ(**R**) and rewrite it into a diffusion equation of another function:

5 $$\frac{\partial f({\bf{R}},\tau )}{\partial \tau }=\frac{{\hslash }^{2}}{2m}{\nabla }^{2}f({\bf{R}},\tau )-{\boldsymbol{\nabla }}\cdot [{\bf{V}}({\bf{R}})f({\bf{R}},\tau )]-[E({\bf{R}})-{E}_{{\rm{R}}}]f({\bf{R}},\tau ),$$

where *f*(**R**, *τ*) = Φ(**R**)Ψ(**R**, *τ*) can be interpreted as a distribution function of the particles at position **R** and imaginary time *τ* if *f*(**R**, *τ*) does not become negative. Here *E*(**R**) = *H*Φ(**R**)/Φ(**R**) is the variational local energy and ${\bf{V}}({\bf{R}})={\hslash }^{2}{\boldsymbol{\nabla }}{\rm{ln}}\,| \Phi ({\bf{R}})|$/*m* can be viewed as a drift velocity of *f*(**R**, *τ*) in the configuration space defined by all possible **R**.

It is clear from Eq. (5) that the time evolution of *f*(**R**, *τ*) is decided by three factors, a pure diffusion term involving ∇^2^, a drift term involving **V**, and a creation/annihilation term involving *E*(**R**) − *E*_R_. The new configuration after a time step Δ*τ* is thus given by

6 $${{\bf{R}}}^{{\prime} }={\bf{R}}+\Delta \tau {\bf{V}}({\bf{R}})+{\boldsymbol{\xi }},$$

where ***ξ*** is a 3*N*-dimensional Gaussian random number with the same variance in each component.

What is left is a contribution that either increase or reduce the population of configurations in the ensemble according to the creation/annihilation rate

7 $${W}_{{\rm{B}}}({\bf{R}})\propto {e}^{-\Delta \tau [E({\bf{R}})-{E}_{{\rm{R}}}]}.$$

The lower the variational local energy *E*(**R**) is, the higher the rate for configuration **R**. This contribution can be sampled by a *branching* process: *M*_B_ ∝ *W*_B_(**R**) copies of the configuration **R** are put into the new ensemble and a mechanism, which does not influence the relative weight of each configuration, needs to be devised to control the overall number of configurations in the ensemble. Usually this control is done by adjusting the reference energy

8 $${E}_{{\rm{R}}}\to {E}_{{\rm{R}}}+\kappa \ {\rm{ln}}\,\frac{{N}_{{\rm{P}}}}{{N}_{{\rm{E}}}}$$

during the simulation with *N*_P_ being the preferred population targeted from a current population of *N*_E_. The small parameter *κ* is selected to control the speed of adjustment. The adjusted reference energy approaches the ground-state energy of the system as the simulation progresses. The first two terms in Eq. (5) are sampled by Eq. (6) and the third term is sampled by Eq. (7) with the overall size of the ensemble controlled by Eq. (8); more detail on the method is available^14^.

For a boson system, function *f*(**R**, *τ*) can always be made real and positive and behaves as a true distribution. Simulation errors still arise from the finite time step and the finite number of ensembles sampled. In principle, the guide wavefunction does not affect the outcome of the simulation if the simulation is performed for a long time and if the guide wavefunction has a finite overlap with the actual ground state of the system. In practice, the overlap between the guide wavefunction and the true ground state impacts the speed of convergence of the simulation, and therefore is an important aspect to consider. For a fermion system, the wavefunction has a nodal structure that is usually unknown and further approximations have to be made because of the complexity of the fermion-sign problem^15^.

Although the reference energy approaches the ground-state energy of the system if the simulation converges, we do not use it to estimate the ground-state energy because it is adjusted during the simulation. Instead the ground-state energy is sampled from the distribution function *f*(**R**, *τ*) at large time *τ*. Because

9 $$\mathop{{\rm{lim}}\,}\limits_{\tau \to \infty }f({\bf{R}},\tau )=\Phi ({\bf{R}}){\Psi }_{0}({\bf{R}}),$$

the ground-state energy can be obtained from the time-dependent variational energy

10 $$E(\tau )=\frac{\langle \Phi | H| \Psi \rangle }{\langle \Phi | \Psi \rangle }=\frac{\int \,E({\bf{R}})f({\bf{R}},\tau )\ d{\bf{R}}}{\int \,f({\bf{R}},\tau )\ d{\bf{R}}},$$

which can be interpreted as an average sampled over the distribution function *f*(**R**, *τ*). For example, if we have an ensemble of configurations given by position vectors ${{\bf{R}}}_{1},{{\bf{R}}}_{2},\ldots ,{{\bf{R}}}_{{N}_{{\rm{E}}}}$, distributed according to *f*(**R**, *τ*), the average energy is given by

11 $$E(\tau )=\frac{\mathop{\sum }\limits_{i=1}^{{N}_{{\rm{E}}}}E({{\bf{R}}}_{i}){W}_{{\rm{B}}}({{\bf{R}}}_{i})}{\mathop{\sum }\limits_{i=1}^{{N}_{{\rm{E}}}}{W}_{{\rm{B}}}({{\bf{R}}}_{i})},$$

which approaches the exact ground-state energy *E*_0_ at *τ* → ∞.

### Guide wavefunction and particle distribution

The trial or guide wavefunction for a many-body system can be constructed based the nature of the system and the interaction between the particles. Here we construct the guide wavefunction for the *N*-body boson system with a pairwise interaction in a Jastrow form:

12 $$\Phi ({\bf{R}})=\Gamma ({\bf{R}})\Lambda ({\bf{R}}),$$

where Γ(**R**) is for the single particle contribution, given by the Bose statistics,

13 $$\Gamma ({\bf{R}})=\mathop{\prod }\limits_{i=1}^{N}\phi ({r}_{i}),$$

with *ϕ*(**r**_*i*_) for the *i*th particle in the system. The Jastrow factor Λ(**R**) is for the two-body correlation, specifically constructed to deal with the unique interaction of the given system,

14 $$\Lambda ({\bf{R}})=\mathop{\prod }\limits_{i > j=1}^{N}f({r}_{ij}),$$

where *f*(*r*_*ij*_) is for the interaction between the *i*th and *j*th particles. Because we are considering a cluster, we have taken a Gaussian function for the single particle state with $\phi (r)={e}^{-{r}^{2}/{r}_{0}^{2}}$, where *r* is the distance of the given particle away from the center of mass of the system and *r*_0_ is a variational parameter on the order of the cluster radius and can be initially set at *r*_0_ = *N*^1/3^*d*, with *d* ≃ 3.0 Å, the separation of two atoms at the minimum interaction between them. The Jastrow factor is assumed to have an exponential form with *f*(*r*) = *e*^−*u*(*r*)^ to target the helium–helium interaction specifically, where

15 $$u(r)={\left(\frac{b}{r}\right)}^{5}+\frac{{a}^{2}}{{c}^{2}+{r}^{2}},$$

with the first term constructed from the cusp condition that forces the kinetic energy to cancel the divergent potential energy when the separation between any two particles approaches zero and the second term constructed from the phonon contribution at the long wavelength limit, significant for large clusters or bulk helium. The estimates of the rough sizes of the variational parameters *a*, *b*, and *c* can be made^16^ based on some physical arguments.

We want to set up an initial configuration that is close to the final configuration but also flexible enough to change for different *N* if needed. One of the ways is to stack up the particles in a simple cubic form with the lattice spaced as *a*_*x*_ = *a*_*y*_ = *a*_*z*_ = *d*. The total number of sites are constructed from *N*_*x*_ *N*_*y*_ *N*_*z*_ ≥ *N* with *N*_*x*_, *N*_*y*_, and *N*_*z*_ being the total number of lattice sites in the *x*, *y*, and *z* directions, respectively. We can pile up particles line by line and then layer by layer until reaching *N*.

The origin of coordinates is adjusted to the center of mass of the system in the initial configuration and after each Monte Carlo move to remove any drifting of the system as a whole from any inaccuracy of the algorithm. We have checked the code without the readjustment of the center of mass and found the result virtually the same.

### Kinetic energy and atom distribution

The evaluation of the total local energy requires both the evaluation of the potential energy and the evaluation of the kinetic energy of the system. The potential energy of the whole system here is just a sum of the *N*(*N* − 1)/2 interaction terms pair by pair. A straightforward evaluation of the kinetic energy of each particle is also possible simply by carrying out the Laplace operator and the total kinetic energy of the system comes from the sum of the contributions from all the particles. There is, however, a better way of evaluating the kinetic energy by split the Laplace operator into two separate terms in the simulation to minimize the fluctuation from one Monte Carlo step to another. The local kinetic energy of each particle in the system can be decomposed as

16 $$-\frac{{\hslash }^{2}}{2m}\frac{1}{\Phi ({\bf{R}})}{\nabla }_{i}^{2}\Phi ({\bf{R}})=2{T}_{i}-| {{\bf{F}}}_{i}{| }^{2},$$

where

17 $${T}_{i}=-\frac{{\hslash }^{2}}{4m}{\nabla }_{i}^{2}{\rm{ln}}\,| \Phi ({\bf{R}})| $$

and

18 $${{\bf{F}}}_{i}=\frac{\hslash }{\sqrt{2m}}{{\boldsymbol{\nabla }}}_{i}{\rm{ln}}\,| \Phi ({\bf{R}})| =\sqrt{\frac{m}{2{\hslash }^{2}}}{{\bf{V}}}_{i}({\bf{R}}).$$

There are several advantages in the evaluation of the kinetic energy with the combination 2*T*_*i*_ − |**F**_*i*_|^2^. In principle, both *T*_*i*_ and |**F**_*i*_|^2^ converge to the kinetic energy *K*_*i*_; this can be shown through integration by parts. But in practice, each of them carries a certain fluctuation during sampling. The combination minimizes the fluctuations because they carry opposite signs in the combination. Evaluations with *T*_*i*_ and |**F**_*i*_|^2^ separately can provide a check on the convergence and the validity of the average through the combination. Note that **F**_*i*_ is a byproduct of the drifting velocity **V**, which is needed as the drifting contribution to a Monte Carlo move. For the helium clusters studied in this work, we have also carried out the evaluation of the kinetic energy without splitting it into the two terms and found virtual no difference within the variance.

To study the structure of a quantum liquid droplet in detail, we have also carried out the simulation of its size and density profile. The center of mass of the system is kept at the origin of the coordinates in each Monte Carlo step and the cluster size is analyzed from the average radius *R* of the cluster, defined from

19 $${R}^{2}=\frac{1}{N}\mathop{\sum }\limits_{i=1}^{N}{r}_{i}^{2}=\langle {r}_{i}^{2}\rangle ,$$

which is sampled during the simulation. Here *R* measures the average distance of the atoms in the cluster away from its center of mass. For a quantum liquid droplet, *R* is finite and remains constant roughly during the simulation. If the atoms are not held together to be a droplet, that is, in a gas phase, *R* will grow infinitely over time.

The distribution of the atoms in a cluster is better measured from the density profile of the cluster

20 $$\rho (r)=\frac{\Delta N}{\Delta \Omega },$$

where Δ*N* is the number of atoms found in the spherical shell between *r* and *r* + Δ*r* with volume ΔΩ = 4*π*[(*r* + Δ*r*)^3^ − *r*^3^]/3.

We have used a combination of constants in the simulation, *ℏ*^2^/(*mk*) = 12.119 245 8 K Å^2^, where *ℏ* is the Planck constant, *m* is the mass of ^4^He, and *k* is the Boltzmann constant. This combination allows us to have lengths measured in Å and energies in K. The imaginary time (*τ* = *it*/*ℏ*) is measured in K^−1^, which makes *τE*, with *E* being energy, dimensionless.

## Simulation results

Before examining the key question of this work on what causes a cluster to become a quantum liquid droplet, we have made test runs of the simulation code with *N* = 84 and *N* = 128 using the Aziz potential HFD-B3-FCI1^11^, which is one of the most accurate potentials for the helium–helium interaction to date, to test our code. The ground-state energies obtained for the two systems agree very well with the values obtained from previous simulations of others^9^. For the system with *N* = 84, we have found the ground-state energy per atom to be *E*_0_/*N* = −3.42(4) K and for the system with *N* = 128, we have found the ground-state energy per atom to be *E*_0_/*N* = −3.85(4) K. The variance in the parentheses is from the sampling of the simulation. The previous Green’s function Monte Carlo simulation leads to a value of *E*_0_/*N* = −3.4 K for a system of 84 atoms^9^. In addition, we have also evaluated the radius $R=\sqrt{\langle {r}_{i}^{2}\rangle }$ for each of the two clusters under the Aziz potential and found *R* = 8.44(3) Å for the system with *N* = 84 and *R* = 9.56(3) Å for the system with *N* = 128.

**Figure 1:**
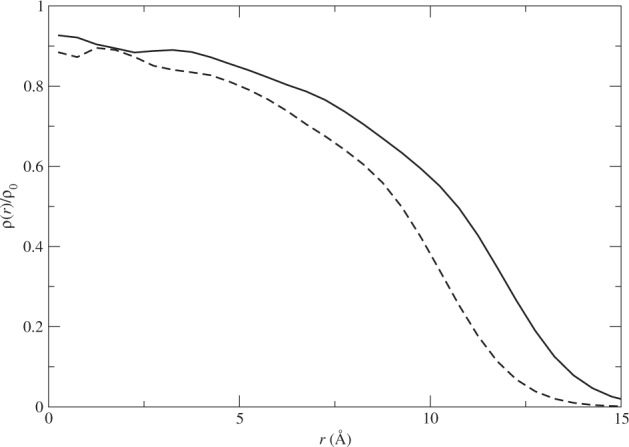
The density profiles of the two sample systems under the Aziz HFD-B3-FCI1 potential with the solid line for *N* = 128 and dashed line for *N* = 84. Here *ρ*_0_ = 0.024 494 Å^3^, the bulk density of ^4^He.

The density profile of a cluster reveals more of its structure than its average radius. In Fig. 1, the density profiles of these two clusters under the Aziz potential are shown. We can see that the density at the center of the cluster is about 90% of the bulk density and drops gradually to zero toward the edge of the clusters. Note that the significant decrease of the density happens around the average radius *R* for each of the clusters. The time step used in this work is Δ*τ* = 0.0001 without the second-order or higher-order correction. The first couple of points of the density near the center of the cluster are less accurate because the small volume elements used in the sampling. The choices made here are adequate to answer our key question and thus no further improvement of accuracy is made. The density profiles also show some shell structures, especially near the center of each cluster.

### The evolution of energy

Our primary goal is to examine the stability a quantum liquid droplet, such as a ^4^He cluster, when the interaction between the particles in the system changes. In order to achieve such a goal, we use the Lennard–Jones potential

21 $$V(r)=4\varepsilon \left[{\left(\frac{\sigma }{r}\right)}^{12}-{\left(\frac{\sigma }{r}\right)}^{6}\right]$$

for the interaction between any two helium atoms. This interaction is less accurate than the better description, such as the Aziz potential, but serves our purpose well because the two contributions to the interaction are clearly separated.

The two parameters in the potential are set differently for different studies and here we use *ε*/*k* = 10.22 K and *σ* = 2.556 Å, the same values used by McMillan in the very first variational quantum Monte Carlo study of liquid ^4^He^13^. The choices reflect the attractive tail of the actual potential between two helium atoms extremely well. Another popular choice^17^ can be found in the studies of helium gases with *ε*/*k* = 10.9 K and *σ* = 2.640 Å.

**Figure 2:**
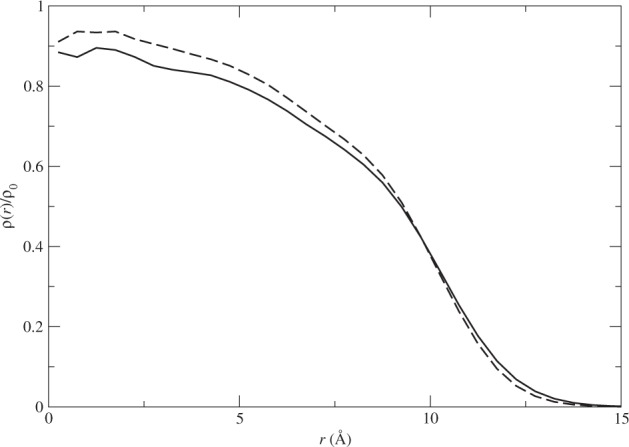
The density profiles of the sample systems with *N* = 84 using the HFD-B3-FCI1 potential (solid line) and the Lennard–Jones potential (dashed line). Here *ρ*_0_ = 0.024 494 Å^3^, the bulk density of ^4^He.

To have a reference on the accuracy of the Lennard–Jones potential used here, we carried out a simulation of ^4^He cluster with *N* = 84 and found the ground-state energy per atom to be *E*_0_/*N* = − 3.15(2) K which is about 8% higher than the corresponding value from the Aziz potential (HFD-B3-FCI1). The average radius of the cluster under the Lennard–Jones potential is *R* = 8.27(3) Å, which is very close to the value of *R* = 8.44(3) Å under HFD-B3-FCI1. We also show here the density profile comparison between the two potentials with *N* = 84 in Fig. 2. Note that the density profiles are very close to each other.

**Figure 3:**
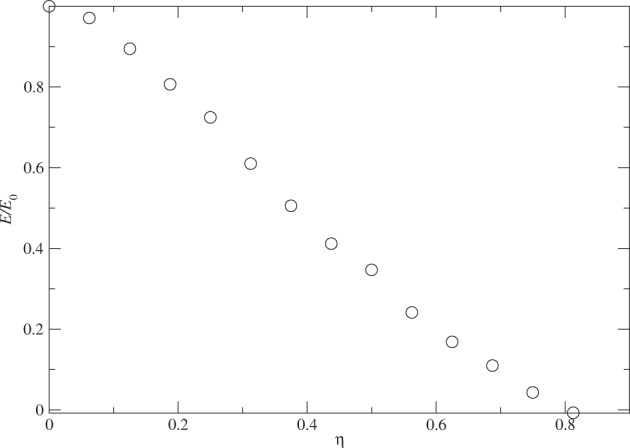
The evolution of the ground-state energy of the cluster of *N* = 128 with *η* = *N*_*x*_/*N*, where *N*_*x*_ is the number of particles that do note have the attractive tail in the interaction among themselves. The energy is normalized by the energy *E*_0_ with *N*_*x*_ = 0.

Now let us turn to the fundamental question: How much attraction must the particles in the cluster have in order to form a quantum liquid droplet? The way we examine this issue is by removing the attractive tail (the second term in the Lennard–Jones potential) in the particle–particle interaction of a selected group of particles in the system. We concentrate on a cluster of 128 helium atoms from now on. Fig. 3 shows the evolution of the ground-state energy per particle in the cluster against the percentage of particles *η* = *N*_*x*_/*N* that do not have the attractive tail in the interaction among themselves.

The data shows that the ground-state energy *E* approaches zero almost linearly in *η* at a value of *η* ≃ 0.8. Note that the total number of pairs of interaction in a system of *N* particles is *N*(*N* − 1)/2. This means that when the system has about half of its interaction pairs without the attractive tail, the cluster is moving away from being a quantum liquid droplet to a gas. To be precise, our diffusion quantum Monte Carlo shows that the total ground-state energy of the cluster *E* approaches zero when *η* = *N*_*x*_/*N* = 104/128 = 0.815, corresponding to a percentage of pairs *N*_*x*_(*N*_*x*_ − 1)/[*N*(*N* − 1)] = 0.66 = 66% that do not have the attractive tail in the interaction. Note that the attractive interaction is removed among *N*_*x*_ atoms but kept among the remaining *N* − *N*_*x*_ atoms and between the two groups.

The energy dependence on *η* appears to be linear except the first point and the last three points. This linear dependence is unexpected because the removal of pairs of attractive interaction is roughly proportional to ${\eta }^{2}\propto {N}_{x}^{2}$. So the number of attractive pairs of interaction also influences the cluster size and reduces the average repulsion between any two atoms in the cluster. The balanced result is a linear dependence between *E* and *η* = *N*_*x*_/*N*.

There are also some interesting details beyond the linear features. The decrease of the energy at small *η* is less than linear and the decrease also slows down a little bit for *η* > 0.5. These questions will be examined further in the future. One source of error for a larger *η* could come from the guide wavefunction, whose single particle part takes the form a bound state, which may not be accurate when the cluster is about to break up, namely, having a phase transition from a quantum liquid droplet to a quantum gas.

### The role of quantum fluctuation

**Figure 4:**
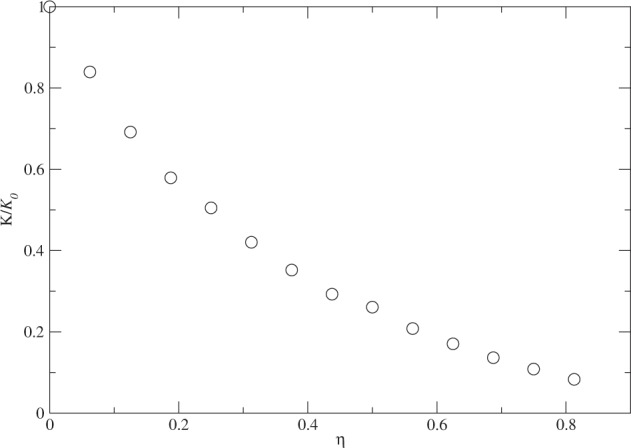
The evolution of the kinetic energy of the cluster of *N* = 128 with *η* = *N*_*x*_/*N*, where *N*_*x*_ is the number of particles that do note have the attractive tail in the interaction among themselves. The kinetic energy is normalized by the kinetic energy *K*_0_ with *N*_*x*_ = 0.

What is happening inside the cluster when the attractive tail of the interaction is removed pair by pair? The ground-state energy shown above has already given us some information about the stability of the quantum liquid droplet. However, the kinetic energy contains more detail on the motion of the particles and reflects the role of quantum fluctuation at zero temperature. A classical system would have zero kinetic energy at absolute zero temperature. In Fig. 4, we show the evolution of the kinetic energy of the cluster with the removal of the attractive tail.

The kinetic energy changes faster than the total ground-state energy when *η* is small and then slows down at about *η* = 0.2. The curve appears to be a quadratic curve of *η* instead of linear. The behavior of the kinetic energy reflects more the size dependence of the motion of the particles. The root-mean-square of the speed of the particle should roughly proportional to the inverse of the cluster size and thus a quadratic dependence on *η* is expected in the kinetic energy if the size of the cluster is close to be linear to *η*.

What is interesting is that the rapid decrease in the kinetic energy at small *η* is not entirely shown in the total ground-state energy because the relevant change in the interaction or potential energy of the particles. The smooth evolution in kinetic energy also indicates the change is gradual and the phase transition from a quantum liquid droplet to a quantum gas is a continuous transition. In other words, the cluster loses its characteristics of a quantum liquid droplet gradually. This may come from the fact the cluster is finite and further study with larger clusters will help clarify the nature of such a phase transition.

### The cluster expansion

**Figure 5:**
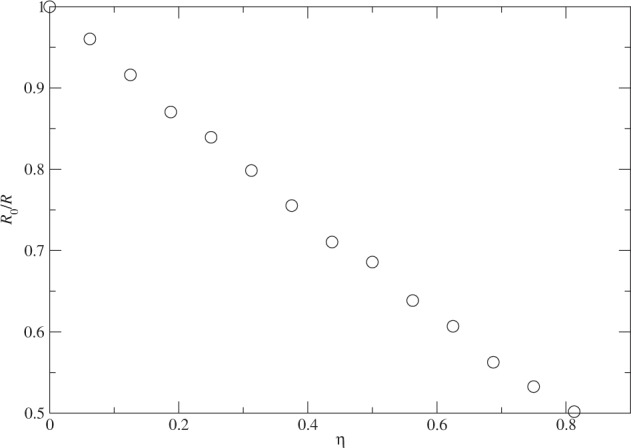
The evolution of the size the cluster of *N* = 128 with *η* = *N*_*x*_/*N*, where *N*_*x*_ is the number of particles that do note have the attractive tail in the interaction among themselves. The size is plotted inversely and normalized by *R*_0_ = *R* at *N*_*x*_ = 0.

It would be nice if we could see the growth of the cluster size matching the unique feature in the kinetic energy. In Fig. 5, we show the relation between the size of the cluster and the removal of the attractive tail in the interaction from the the root-mean-square distance of the particles away from the center of mass of the cluster. The size is plotted inversely and normalized by the corresponding value without removing any attractive tail. Namely, we are examining the relation between *R*_0_/*R* and *η* with *R*_0_ being *R* at *η* = *N*_*x*_/*N* = 0. The entire expansion with *R*_0_/*R* being linear in *η* matches the quadratic behavior of *K* over *η* precisely. The change in *R* again is gradual and indicates a continuous transition of the cluster from a quantum liquid droplet to a gas. Note that when the system is near a gas phase, the guide wavefunction is no longer valid because it does not contain a nonzero component at *r* → ∞. Once again the linear relation between *R*_0_/*R* and *η* is surprising.

**Figure 6:**
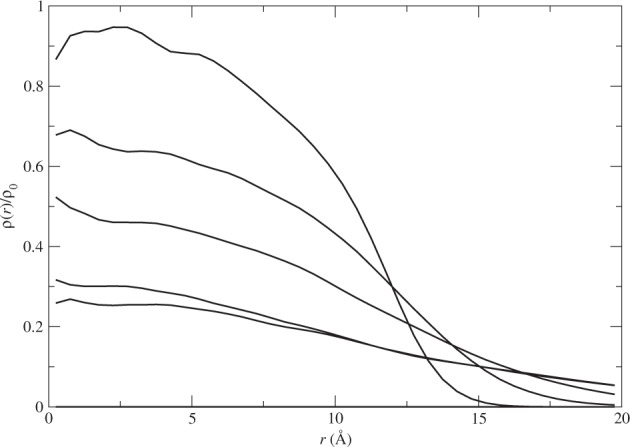
The density profiles of the cluster with *N* = 128 with the increase of the number of atoms *N*_*x*_ losing the attraction among them with *η* = *N*_*x*_/*N* = 0, 0.25, 0.50, 0.75, and 0.8125, in the order of decreasing density at the cluster center *r* = 0. Here *ρ*_0_ = 0.024494 Å^3^ is the bulk density of ^4^He.

To see the growth of the cluster size with *η* in more detail, we show the density profiles of the cluster with different values of *η*. When the attractive tail in the interaction is removed pair by pair, the density profile of the cluster gives us the best measure of the change in the particle distribution in the cluster. In Fig. 6, we show the density profiles of the cluster at *η* = *N*_*x*_/*N* = 0, 0.25, 0.50, 0.75, and 0.8125, respectively.

We see clearly an evolution from a compact cluster, or a quantum liquid droplet with a high density at the center to a gas that has nearly a uniform low density. Specifically, we see a linear decrease of the density at the center with the increase of *η*. This again is a sign of a continuous phase transition from the quantum liquid droplet to a quantum gas.

## Conclusions

What makes a cluster of particles into a quantum liquid droplet? The attractive interaction between the particles is certainly driving force to make a quantum many-body system into a liquid if we want to state it in the simplest form. But this is not the whole story. Our simulation here also shows that a certain amount of attractive interaction is needed in order to overcome the quantum fluctuation at zero temperature. The size of the cluster appeared to be critical in the sense that the kinetic energy of a cluster is proportional inversely to the cluster size and more kinetic energy at zero temperature means higher quantum fluctuation in the system. While the simulation presented in this work shows that removal of two-thirds of pairs of attractive interaction in a cluster of 128 helium atoms causes the cluster to break up, larger systems still need to be explored before we know what will happen to a bulk system, where the biding energy of each particle is higher.

Another aspect of the helium clusters is being examined by our group right now by modifying the relative strength of the attractive tail on all pairs of interaction. We expect that the cluster size will increase with the reduction of the strength of the attractive tail and the result will be reported in a future publication.

The current studies of the formation of quantum liquid droplets from cold atoms concentrate on switching repulsive interaction between some atoms into attractive interaction. In principle, the competition between the attractive interaction and quantum fluctuation is the still primary concern. However, the density of cold atoms is orders of magnitude lower than the density of helium clusters and the number of atoms in a cold atom cluster is several orders of magnitude higher than the number of helium atoms studied here. Thus we do expect some differences show up in these two different systems. One of our current projects is to look into a model of cold atoms and ask specifically these questions.
